# Exploring the Genomic Dynamics of the Monkeypox Epidemic in Paraguay

**DOI:** 10.3390/v16010083

**Published:** 2024-01-04

**Authors:** Cynthia Vazquez, Vagner Fonseca, Andrea Gomez de la Fuente, Sandra Gonzalez, Fatima Fleitas, Mauricio Lima, Natália R. Guimarães, Felipe C. M. Iani, Analia Rojas, Tania Alfonso, Cesar Cantero, Julio Barrios, Shirley Villalba, Maria Jose Ortega, Juan Torales, Maria Liz Gamarra, Carolina Aquino, Leticia Franco, Jairo Mendez Rico, Luiz Carlos Junior Alcantara, Marta Giovanetti

**Affiliations:** 1 Laboratorio Central de Salud Pública, Asunción 001535, Paraguay; cynthiavlm@yahoo.com (C.V.); andre.gff585@gmail.com (A.G.d.l.F.); sanygonz@hotmail.com (S.G.); fatifleitas91@gmail.com (F.F.); amnrojas@gmail.com (A.R.); tania_alfonzo94@hotmail.com (T.A.); cesarcantero24@gmail.com (C.C.); biojulioc@gmail.com (J.B.); shirleyvillalba@hotmail.com (S.V.); majosortega@yahoo.es (M.J.O.); juanbt15@hotmail.com (J.T.); malizga@yahoo.com (M.L.G.); lcala_py@hotmail.com (C.A.); 2 Coordenação de Vigilância, Preparação e Resposta à Emergências e Desastres (PHE), Organização Pan-Americana da Saúde/Organização Mundial da Saúde (OPAS/OMS), Brasilia 70312-970, Brazil; 3 Department of Exact and Earth Sciences, University of the State of Bahia, Salvador 41150-000, Brazil; 4 Laboratorio Central de Saúde Pública do Estado de Minas Gerais, Fundação Ezequiel Dias, Belo Horizonte 30510-010, Brazil; maurili15@hotmail.com (M.L.); natyroguiman@yahoo.com.br (N.R.G.); felipeemrede@gmail.com (F.C.M.I.); 5 Infectious Hazards Management, Health Emergencies Department (PHE), Pan American Health Organization/World Health Organization (PAHO/WHO), Washington, DC 20037, USA; francolet@paho.org (L.F.); ricoj@paho.org (J.M.R.); 6 Instituto Rene Rachou, Fundação Oswaldo Cruz, Belo Horizonte 30190-002, Brazil; 7 Climate Amplified Diseases and Epidemics (CLIMADE), University of Washington, Seattle, WA 98195, USA; 8 Oswaldo Cruz Foundation (FIOCRUZ), Salvador, Bahia 40296-710, Brazil; 9 Sciences and Technologies for Sustainable Development and One Health, University of Campus Bio-Medico, 00128 Rome, Italy

**Keywords:** monkeypox virus, mpox, genomic surveillance, Paraguay

## Abstract

In recent months, Paraguay has been grappled with a notable monkeypox outbreak, straining its healthcare infrastructure. The sudden spike in cases underlines the imperative need for a comprehensive understanding of the virus’s dynamics, enabling the formulation of robust containment measures. To address this challenge, our team joined forces with the Central Public Health Laboratory of Asunción and the Pan-American Health Organization. Through this collaboration, we employed portable whole-genome sequencing combined with phylodynamic analysis to examine the MPXV strains circulating in Paraguay. Our genomic monitoring approach has produced the first 30 whole-genome sequences from Paraguay, all of which were identified under lineage IIb. Interestingly, our data suggest that the origin of the monkeypox virus in Paraguay at the beginning of 2022 can be traced back to Brazil. This introduction subsequently catalyzed further community spread that was further exacerbated by several independent introduction events as time progressed. These findings not only shed light on the transmission patterns of the virus but also highlight the pivotal role such insights play in sculpting effective response strategies and driving impactful public health measures. Furthermore, our findings strongly advocate intensified surveillance at international borders, ensuring swift detection and proactive countermeasures against potential outbreaks in the future.

## 1. Introduction

Zoonotic monkeypox is an infection transmitted by the monkeypox virus (MPXV), a double-stranded DNA virus classified within the *Orthopoxvirus* genus [1]. Human transmission of the virus predominantly occurs via close contact with the respiratory secretions, cutaneous lesions (lesion crusts), or blood, bodily fluids, or lesions of an infected individual [2]. The clinical manifestations of monkeypox can be divided into two distinct phases. The first phase, known as the prodromal period, lasts for 1–4 days and is characterized by non-specific symptoms such as fever, headaches, and exhaustion [2]. This is followed by the second phase, known as the skin rash phase, which normally begins 1–3 days after the initial onset of fever. Lymphadenopathy, often occurring simultaneously with the skin rash, can also manifest during the prodromal phase. The enlarged lymph nodes have a distinctively solid texture, sensitivity, and occasional discomfort. The total duration of these symptoms often ranges from 2 to 5 weeks [2]. The MPXV genomic diversity might be categorized into two taxonomic groups: Clade I, formerly known as the Central African (or Congo Basin) clade, and Clade II, previously referred to as the West African clade [2].

Although primarily found in West and Central Africa, cases of Monkeypox have also been reported in individuals who have traveled from these regions. 

In 2022, an atypical surge of monkeypox cases was observed in areas not previously known to be endemic. These were associated with a specific sub-clade of Clade II, namely, lineage B.1 [3]. Remarkably, these cases affected individuals with no direct contact history with the traditionally known endemic regions for the disease’s transmission. These cases affected individuals who had not been in contact with regions traditionally associated with the disease’s transmission. As a result, the World Health Organization Director-General defined the multi-country monkeypox outbreak a Public Health Emergency of Inter-national Concern on 23 July 2022 [4]. 

Amid this global concern, Paraguay has recently faced a monkeypox outbreak, prompting heightened alertness and action from its health officials. The initial recorded instance of monkeypox in Paraguay occurred on 24 August 2022, when a traveler, who had recently returned from Brazil, tested positive for the disease. This positive diagnosis was subsequently confirmed at the national health laboratory through molecular screening using real-time quantitative polymerase chain reaction (RT-qPCR) [5]. In response to this, the national health authorities promptly took action, implementing measures such as intensified surveillance, contact tracing, and isolation protocols. As of July 2023, which corresponds with the time of this study, the number of cases confirmed through molecular screening have risen sharply to a total of 125 [5]. Recognizing the importance of international collaboration, Paraguay sought support from the Pan-American Health Organization (PAHO) to strengthen its response efforts. However, despite these efforts, there are still significant gaps in understanding the genomic diversity and evolution of the circulating MPXV lineages in Paraguay. To address this knowledge gap, in this study, we employed next-generation sequencing to generate 30 new genomes, providing valuable preliminary insights into the introduction and spread of MPXV within the country.

## 2. Materials and Methods

From August 2022 to November 2023, clinical samples were collected from individuals presenting with symptoms of MPXV, such as fever, myalgias, headache, rash, and lesions (including crusts). These samples were then forwarded to the Central Public Health Laboratory of Asunción for molecular testing. Genetic material from the exudate of lesions obtained from affected patients was isolated for detailed molecular screening using RT-qPCR. Positive samples (*n* = 30) underwent a process of amplification of the entire genome using Q5 High-Fidelity Hot-Start DNA Polymerase (New England Biolabs, MA, USA). The amplification process was initiated using a set of primers specifically designed for MPXV as recently described by [6]. After the amplification process, the resulting DNA amplicons were subjected to a purification phase. Purifications were conducted using AMPure XP beads (Beckman Coulter, Brea, CA, USA). The COVIDseq Kit (Illumina, San Diego, CA, USA) was used to prepare genomic libraries. Originally designed for SARS-CoV-2 genomic research, this kit has recently demonstrated its versatility and effectiveness in many viral genomic applications [6]. It enables an efficient procedure for preparing libraries that are ready for high-throughput sequencing. The selection of samples for sequencing was guided by criteria such as Ct values of less than 35 and the availability of epidemiological metadata. These metadata encompass symptom onset date, sample collection date, patient demographics (sex, age, municipality), symptoms, comorbidities, and disease classification. Sequencing was conducted on the Illumina MiSeq platform (Illumina, San Diego, CA, USA), in accordance with the manufacturer’s guidelines. The Genome Detective software Version 2.72 [7] was utilized to acquire consensus sequences. The process of determining the genotype was carried out utilizing the Nextclade web program [8]. In order to study the evolution of MPXV in Paraguay, we merged the recently obtained full genome sequences (*n* = 30) with the representative globally accessible sequences obtained from GISAID (*n* = 1428) until 10 July 2022. The sequences were aligned using the MAFFT software version 7.520 [9] and further edited to eliminate any biological artifacts using Aliview [10]. The GTR nucleotide substitution model, determined as the most suitable model by the ModelFinder application in IQ-TREE2 [11], was employed to calculate maximum likelihood (ML) phylogenetic trees. The tree topology was assessed by utilizing 1000 bootstrap replicates. The presence of a temporal signal was evaluated using TempEst v.1.5.3 [12], and time-scaled phylogenetic trees were inferred using the BEAST software v.1.10.4 [13]. In order to determine the most suitable molecular clock model for the Bayesian phylogenetic analysis, we employed a rigorous model selection technique that incorporated both path-sampling (PS) and steppingstone (SS) procedures [14]. The uncorrelated relaxed molecular clock model was selected for all datasets by evaluating marginal likelihoods using the codon-based SRD06 model of nucleotide substitution and the nonparametric Bayesian Skyline coalescent model. The MCMC analyses were conducted using BEAST v1.10.4. The analyses were duplicated and ran for 20 million iterations, with samples taken every 10,000 steps in the chain. Tracer was employed to evaluate the convergence of each run, ensuring that the effective sample size for all pertinent model parameters exceeded 200. Following the removal of the initial 10% as burn-in, MCC trees were summarized for each run using TreeAnnotator v.1.10.4. 

## 3. Results

**Figure 1:**
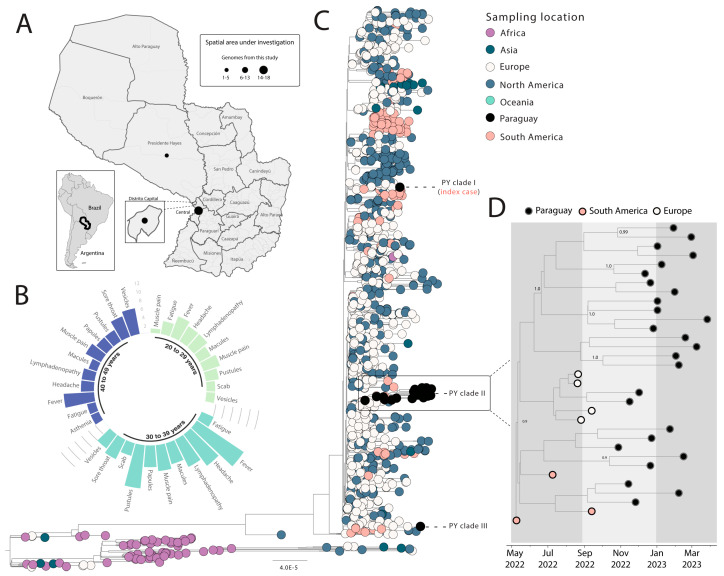
Spatial and temporal distribution of MPXV cases in Paraguay. (**A**) Map of Paraguay displays the number of MPXV genome sequences per department, with circle size representing the number of new genomes generated in this study. (**B**) Symptom frequency among 30 confirmed MPXV infection cases. (**C**) Maximum likelihood tree of MPXV whole-genome sequences assigned to Clade IIb, including 30 genomes from this study and 1428 globally representative reference strains. Tips are color-coded based on sample source location. (**D**) Time-scaled maximum clade credibility tree of the Paraguay Clade II (PY Clade 2), including 26 new genomes from Paraguay and 7 reference strains from European and South American countries. Tips are color-coded based on sample source location, and values around nodes represent posterior probability support inferred using Bayesian Evolutionary Analysis with a molecular clock approach.

Illumina Miseq sequencing was performed on selected MPXV-positive samples (*n* = 30) which showed sufficient DNA (≥2 ng/μL) for library preparation. The average cycle threshold (Ct) values for PCR of those positive samples were 20.20 (range: 16.4 to 26.7). Table 1 provides the additional epidemiological details of the processed samples. The sequencing process resulted in an average coverage of 98%. The novel strains were obtained from three different districts (Figure 1A). 

In terms of age distribution, the median age of infected individuals was 36 years, ranging from 21 to 47 years, with 97% (*n* = 30) being male (Table 1). 

Among the patients whose samples were sequenced, 37.0% (11/30) were identified as HIV-positive. A diverse range of signs and symptoms were observed across various age classes with the most frequently reported being fever (25/30), pustules (16/30), headache (16/30), and muscle pain (15/30) as depicted in Figure 1B. Intriguingly, of the 30 patients, only a single individual, who turned out to be the initial imported case in the nation, had disclosed a recent travel history to Brazil.

To further understand the phylodynamics of MPXV in the region, an in-depth analysis was undertaken. This analysis incorporated the 30 newly sequenced samples along with 1428 globally referenced strains from the MPXV lineage IIb, which were obtained from GISAID (Figure 1C). The results revealed the presence of three distinct clades among the recent isolates, i.e., PY Clade I (including the first imported case belonging to a returning traveler from Brazil), Clade II, and Clade III, as illustrated in Figure 1C. This diversity underscores the potential for regional transmission dynamics and the necessity of ongoing surveillance to monitor the evolution and spread of the virus. The presence of different clades suggests that multiple independent introduction events have occurred over time within the country. Notably, Clade II emerged as a robust cluster within the phylogenetic analysis (Figure 1C), indicating a potential for sustained local transmission. To delve deeper into the evolutionary dynamics of Clade II, we focused on a smaller dataset consisting of 26 sequences from Paraguay, along with 7 reference strains from European and South American countries belonging to this clade. Using this subset, we constructed a dated phylogeny, indicating a probable origin around early June 2022, with a mean time estimation and a 95% highest posterior density (HPD) range from late May 2022 to early August 2022 (Figure 1D).

## 4. Discussion

During 2022–2023, the world witnessed a widespread outbreak of mpox, primarily attributed to a strain known as Clade IIb. This outbreak saw rapid global dissemination, resulting in approximately 87,000 cases and 112 fatalities across 110 countries.

In the context of this reemergence, this study analyzed the first 30 whole-genome sequences of the MPXV isolated in Paraguay. The aim was to explore the evolutionary dynamics and patterns of transmission of the monkeypox virus on a national scale. Such analysis is critical for understanding the behavior of the virus and the nature of the outbreak within the country. Our findings revealed that all newly sequenced strains from Paraguay belonged to lineage IIb. The detection of this lineage, previously identified in several African countries, highlights the expansive geographical reach of lineage IIb, signifying its spread well beyond the African continent. Our analysis additionally reveals that the initial case of MPXV in Paraguay in early 2022 can be traced back to Brazil, resulting in subsequent local transmissions influenced by multiple independent introduction events as evidenced by the detection of three independent clades (labelled as PY clades I-III). Our data further indicate that Clade II emerged as a robust cluster, highlighting a likely sustained local transmission throughout the country. These introductions, potentially driven by increased human mobility, underscore the concerning threat of introducing novel and re-emerging strains into areas that have previously remained unaffected by the virus. Delving deeper into understanding the origins of these introductions is pivotal, as it can inform and shape national as well as international health policies and interventions.

Together these results contribute to the genomic surveillance of MPXV in Paraguay, emphasizing the crucial role of a prompt response in effectively containing the spread of emerging and re-emerging viral threats with epidemic or pandemic potential. By leveraging genomic surveillance, a proactive approach can promptly identify and respond to viral threats, minimizing their impact on public health.

## Figures and Tables

**Table 1:** Epidemiological data of the 30 MPXV samples sequenced as a part of this study.

ID	Clade	Lineage	Ct	Coverage	District	Collection Date	Date of the Onset Symptom	Gender	Age	GISAID-ID
hMPXV|Paraguay|585623|2023-01-23	IIb	B.1	19.4	98.8	Presidente Hayes	2023-01-23	2023-01-16	M	29	EPI_ISL_17988349
hMPXV|Paraguay|590800|2023-01-29	IIb	B.1	21.2	97.9	Central	2023-01-29	2023-01-21	M	31	EPI_ISL_17988350
hMPXV|Paraguay|591628|2023-01-31	IIb	B.1	19	98.1	Capital	2023-01-31	2023-01-22	M	40	EPI_ISL_17988351
hMPXV|Paraguay|593520|2023-02-02	IIb	B.1	22.6	98.8	Central	2023-02-02	2023-01-23	M	35	EPI_ISL_17988352
hMPXV|Paraguay|595642|2023-02-07	IIb	B.1	21.2	97.7	Capital	2023-02-07	2023-02-01	M	38	EPI_ISL_17988353
hMPXV|Paraguay|595736|2023-02-07	IIb	B.1	19.8	97.5	Capital	2023-02-07	2023-01-29	M	34	EPI_ISL_17988354
hMPXV|Paraguay|601896|2023-02-15	IIb	B.1	21.6	96.3	Central	2023-02-15	2023-02-08	F	47	EPI_ISL_17988355
hMPXV|Paraguay|603652|2023-02-16	IIb	B.1	19.6	97.1	Capital	2023-02-16	2023-02-10	M	41	EPI_ISL_17988356
hMPXV|Paraguay|607376|2023-02-18	IIb	B.1	21.3	97.7	Capital	2023-02-18	2023-02-14	M	43	EPI_ISL_17988357
hMPXV|Paraguay|613691|2023-02-28	IIb	B.1	19.8	98	Capital	2023-02-28	2023-02-25	M	37	EPI_ISL_17988358
hMPXV|Paraguay|617799|2023-03-03	IIb	B.1	16.5	98.5	Capital	2023-03-03	2023-02-26	M	37	EPI_ISL_17988359
hMPXV|Paraguay|621397|2023-03-09	IIb	B.1	18.5	98.6	Central	2023-03-09	2023-03-02	M	31	EPI_ISL_17988360
hMPXV|Paraguay|635677|2023-03-27	IIb	B.1	20.8	97.2	Central	2023-03-27	2023-03-22	M	43	EPI_ISL_17988361
hMPXV|Paraguay|726874|2022-08-24	IIb	B.1	21.4	99.2	Central	2022-08-24	2022-08-15	M	35	EPI_ISL_17988362
hMPXV|Paraguay|737804|2022-10-28	IIb	B.1	19.7	98.1	Central	2022-10-28	2022-10-28	M	30	EPI_ISL_17988363
hMPXV|Paraguay|739989|2022-11-14	IIb	B.1	19.8	99.2	Capital	2022-11-14	2022-11-14	M	27	EPI_ISL_17988364
hMPXV|Paraguay|740405|2022-11-16	IIb	B.1	17.4	99.1	Central	2022-11-16	2022-11-09	M	24	EPI_ISL_17988365
hMPXV|Paraguay|741084|2023-11-20	IIb	B.1	16.4	96.8	Central	2023-11-20	2022-11-05	M	44	EPI_ISL_17988366
hMPXV|Paraguay|742315|2022-11-26	IIb	B.1	17	98	Central	2022-11-26	2022-11-22	M	44	EPI_ISL_17988367
hMPXV|Paraguay|743529|2022-12-02	IIb	B.1	18.1	96.9	Central	2022-12-02	2022-11-25	M	42	EPI_ISL_17988368
hMPXV|Paraguay|744881|2022-12-12	IIb	B.1	19.5	97.6	Capital	2022-12-12	2022-12-05	M	44	EPI_ISL_17988369
hMPXV|Paraguay|745948|2022-12-21	IIb	B.1	18.4	99.4	Capital	2022-12-21	2022-12-19	M	23	EPI_ISL_17988370
hMPXV|Paraguay|746039|2022-12-23	IIb	B.1	17.6	97.6	Central	2022-12-23	2022-12-19	M	41	EPI_ISL_17988371
hMPXV|Paraguay|746338|2022-12-26	IIb	B.1	21.2	98.9	Central	2022-12-26	2022-11-18	M	42	EPI_ISL_17988372
hMPXV|Paraguay|746914|2022-12-21	IIb	B.1	23.1	95.9	Capital	2022-12-21	2023-12-21	M	34	EPI_ISL_17988373
hMPXV|Paraguay|747004|2023-01-02	IIb	B.1	22.01	96.2	Central	2023-01-02	2022-12-29	M	36	EPI_ISL_17988374
hMPXV|Paraguay|747005|2023-01-02	IIb	B.1	22.1	97	Central	2023-01-02	2022-12-27	M	32	EPI_ISL_17988375
hMPXV|Paraguay|747006|2023-01-02	IIb	B.1	21.3	97	Capital	2023-01-02	2022-12-26	M	21	EPI_ISL_17988376
hMPXV|Paraguay|747046|2023-01-02	IIb	B.1	26.7	95.6	Capital	2023-01-02	2022-12-31	M	31	EPI_ISL_17988377
hMPXV|Paraguay|747536|2023-01-09	IIb	B.1	22.8	97.2	Central	2023-01-09	2022-12-20	M	38	EPI_ISL_17988378

## Data Availability

Newly generated MPXV sequences have been deposited in GISAID under accession numbers: EPI_ISL_17988349-EPI_ISL_17988378.
